# An ADPLL-Based GFSK Modulator with Two-Point Modulation for IoT Applications

**DOI:** 10.3390/s24165255

**Published:** 2024-08-14

**Authors:** Nam-Seog Kim

**Affiliations:** Department of Information and Communication Engineering, School of Electrical and Computer Engineering, Chungbuk National University, Cheongju-si 28644, Republic of Korea; namseog.kim@cbnu.ac.kr

**Keywords:** all-digital phase-locked loop (ADPLL), Bluetooth low energy (BLE), digitally controlled oscillator (DCO), frequency shift keying (FSK) error, frequency tuning range, Gaussian frequency shift keying (GFSK), Internet of Things (IoT), least mean square (LMS) algorithm, low dropout regulator (LDO), phase noise, process, voltage, and temperature (PVT) variations, stable modulation index (SMI), time-to-digital converter (TDC), two-point modulation (TPM), wireless sensor networks (WSNs)

## Abstract

To establish ubiquitous and energy-efficient wireless sensor networks (WSNs), short-range Internet of Things (IoT) devices require Bluetooth low energy (BLE) technology, which functions at 2.4 GHz. This study presents a novel approach as follows: a fully integrated all-digital phase-locked loop (ADPLL)-based Gaussian frequency shift keying (GFSK) modulator incorporating two-point modulation (TPM). The modulator aims to enhance the efficiency of BLE communication in these networks. The design includes a time-to-digital converter (TDC) with the following three key features to improve linearity and time resolution: fast settling time, low dropout regulators (LDOs) that adapt to process, voltage, and temperature (PVT) variations, and interpolation assisted by an analog-to-digital converter (ADC). It features a digital controlled oscillator (DCO) with two key enhancements as follows: ΔΣ modulator dithering and hierarchical capacitive banks, which expand the frequency tuning range and improve linearity, and an integrated, fast-converging least-mean-square (LMS) algorithm for DCO gain calibration, which ensures compliance with BLE 5.0 stable modulation index (SMI) requirements. Implemented in a 28 nm CMOS process, occupying an active area of 0.33 mm^2^, the modulator demonstrates a wide frequency tuning range of from 2.21 to 2.58 GHz, in-band phase noise of −102.1 dBc/Hz, and FSK error of 1.42% while consuming 1.6 mW.

## 1. Introduction

The Internet of Things (IoT) has emerged as a transformative paradigm, interconnecting a vast array of devices to create intelligent, responsive systems across various sectors. At the heart of this revolution are wireless sensor networks (WSNs), which form the foundational infrastructure for IoT applications. WSNs enable the collection and transmission of data from the physical world, making them indispensable for realizing the full potential of IoT. Within the broader category of WSNs, short-range wireless sensor networks have gained particular prominence in IoT applications. These networks are crucial in scenarios where devices need to communicate over limited distances, such as in smart homes, industrial automation, healthcare monitoring, and personal area networks. The proliferation of the IoT has intensified the demand for short-range WSNs that can support a wide array of applications, including home and industrial automation, security systems, patient monitoring, process control, consumer electronics, and fitness tracking [1]. To meet the diverse needs of IoT applications, short-range WSNs require communication technologies that are low-power, cost-effective, and capable of high data rates. These requirements have driven the development and adoption of specialized protocols and standards. Among these, Bluetooth low energy (BLE) has emerged as a dominant force within the IoT ecosystem [2].

BLE, designed to streamline device pairing and simplify the complex discovery protocols of classic Bluetooth, is particularly well-suited for short data exchanges in IoT contexts [3]. Its integration into smartphones and mobile devices has positioned BLE favorably in the growing IoT landscape. As IoT deployments expand, experts anticipate a significant increase in BLE-enabled devices, particularly in environments like retail stores where large numbers of BLE tags may simultaneously advertise products and services [2]. The efficiency and reliability of BLE communication hinge on its underlying modulation scheme, Gaussian frequency-shift keying (GFSK). GFSK offers a balance of energy efficiency, spectral efficiency, and robust communication, making it well suited to the needs of short-range WSNs in IoT applications [4]. By varying the frequency of a carrier signal according to the input data and applying Gaussian filtering to smooth these frequency shifts, GFSK enables BLE to achieve reliable data transmission while maintaining low power consumption—a critical factor for battery-operated IoT devices [4]. As IoT applications continue to evolve, demanding higher data rates and more efficient spectrum utilization, there is a growing need for advanced GFSK modulator designs that can meet these challenges while complying with the latest BLE specifications. This paper presents a novel approach to GFSK modulation for IoT applications, focusing on enhancing the efficiency and performance of BLE communication in short-range WSNs.

The modulation index is a key parameter that defines the extent of frequency deviation in the GFSK modulation scheme in the BLE. Specifically, the modulation index (*h*) is the ratio of the frequency deviation to the data rate as Equation (1) [5].

(1) $$h=\frac{f_{dev}}{2R_{b}}\;$$

where *f_dev_* is the frequency deviation and *R*_*b*_ is the bit rate. The modulation index of the BLE typically ranges between 0.45 and 0.55. This range is selected to balance the trade-offs between bandwidth efficiency and error performance. Then, the *f_dev_* is between 450 and 550 kHz at a 2 Mb/s data rate, which is a 10% variation from the ideal modulated frequency. A modulation index of around 0.5 is ideal as it helps in achieving a good balance between the signal-to-noise ratio (SNR) and the bandwidth used. However, the BLE version 5.1 specification adds transmitter specifications for the angle of arrival (AoA) and angle of departure (AoD) to identify the orientation of a BLE module with improved accuracy [6]. This is defined by the stable modulation index (SMI), where the *f_dev_* is between 495 and 505 kHz at a 2 Mb/s data rate, with a 1% variation from the ideal modulation frequency.

**Figure 1:**
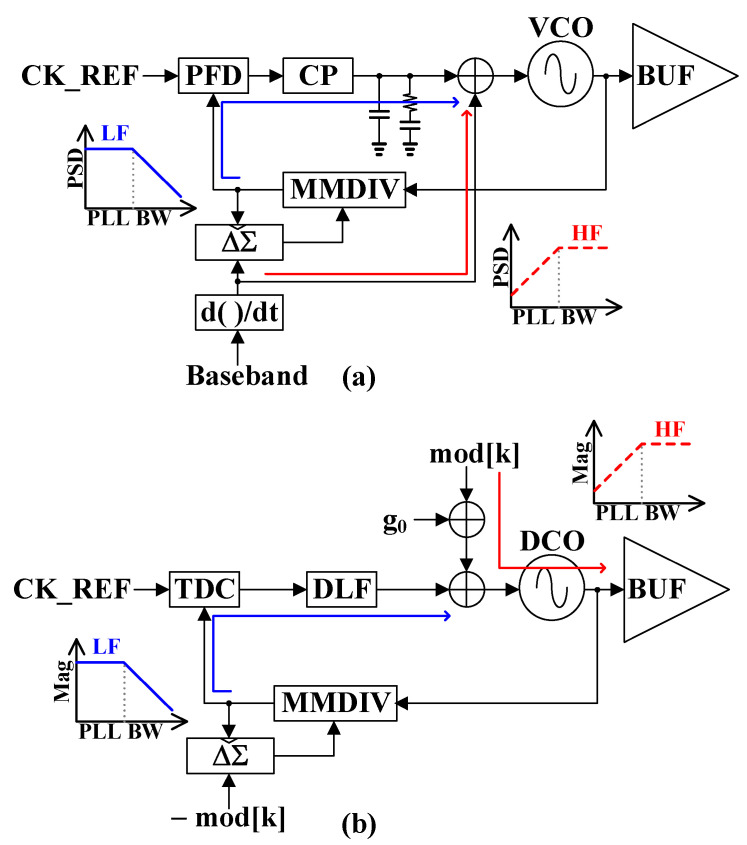
Two-point modulation with (**a**) conventional charge pump PLL and (**b**) all digital PLL.

In most state-of-the-art systems, the GFSK is implemented by modulating the PLL’s output frequency during closed-loop operation, which means that the PLL’s bandwidth is a limiting factor concerning the maximum data rate that may be applied. Two-point modulation (TPM) has been investigated as a method to enable data rates exceeding the bandwidth of the conventional charge pump phase-locked loop (CPPLL), as shown in Figure 1a [7]. The low-frequency components of the baseband transmit signal are frequency-modulated within the PLL bandwidth through a fractional-N frequency synthesizer, while the high-frequency components are frequency-modulated outside the PLL bandwidth by applying the baseband transmit signal directly to the second tuning input of the voltage-controlled oscillator (VCO). This scheme can resolve the problem of the modulation bandwidth being limited to the PLL bandwidth. Therefore, it can not only maintain high data rate modulation within a closed loop, but can also exclude the use of mixers and RF band-pass filters. However, in deep sub-micron CMOS technology, the CPPLL encounters limited voltage headroom, current mismatch, filter capacitor leakage, and reduced dynamic range in nano-scale CMOS processes.

All-digital phase-locked loops (ADPLLs) shown in Figure 1b circumvent these issues with CPPLLs and benefit from a higher level of integration and enhanced programmability [8]. The TPM is constructed by injecting modulation signal mod[k] to the MMDIV for the low-pass transfer function and the DCO for the high-pass transfer function simultaneously to support wideband modulation. Thus, the ADPLL-based modulation is seen as a viable solution for achieving the TPM. However, the ADPLL requires a highly linear TDC with high time resolution. Otherwise, TDC nonlinearity can introduce fractional spurs and degrade in-band phase noise in the ADPLL. DCO nonlinearity degrades phase noise performance, distorts GFSK modulated signals, and affects the modulation index. Moreover, the delay and gain mismatches between the high-pass modulation path and the low-pass modulation path are critical factors in TPM [9,10,11,12,13].

This paper introduces an ADPLL-based GFSK modulator with TPM to support BLE 5.0 specifications. The TDC has high linearity along with phase error in the feedback loop by using a PVT-adaptive LDO and ADC-assisted high time resolution. The DCO utilizes a ΔΣ modulator for dithering and linearized capacitive banks to improve linearity, and a wide tuning range is achieved by using coarse/fine hierarchical capacitive banks. A proposed LMS-based DCO gain calibration meets 2 Mb/s data rate GFSK modulation and SMI specifications of BLE 5.0. This article is organized in the following manner. Section 2 presents an architecture of the ADPLL-based GFSK modulator. Section 3 describes the overall circuitry of the proposed modulator. Section 4 discusses the experimental results of the proposed GFSK modulator obtained on the integrated circuit fabricated in 28 nm CMOS technology. Finally, the main contributions are summarized in Section 5.

## 2. Related Works

Recent years have seen significant advancements in the development of efficient GFSK modulators for BLE applications in IoT contexts. This section provides a comprehensive overview of key contributions and approaches from the recent literature, highlighting the evolution of techniques and identifying areas for potential improvement.

All-digital phase-locked loop (ADPLL) architectures have gained prominence in BLE transmitter designs due to their programmability and suitability for advanced CMOS processes. Ding et al. [14] demonstrated an ADPLL-based Bluetooth 5 transceiver in 40 nm CMOS, achieving a 2 Mb/s data rate with a phase-tracking receiver. Chen et al. [15] proposed an ultra-low-power BLE transmitter using a ring oscillator-based ADPLL, showcasing significant power reductions in digital designs. TPM has emerged as a key technique to overcome PLL bandwidth limitations in high-speed modulation. Huang et al. [16] implemented a two-point modulation scheme in their sub-2.4 GHz transmitter, achieving a 2.3% FSK error, demonstrating the effectiveness of TPM in improving modulation accuracy. Digitally controlled oscillator (DCO) linearity and calibration are critical for ADPLL performance. Liu et al. [17] presented a GFSK modulator based on 16 bang-bang DPLLs without background digital calibration, achieving a 2.86% EVM. Staszewski et al. [18] introduced an LMS-based calibration of an RF digitally controlled oscillator, providing a foundation for adaptive calibration techniques. Time-to-digital converters (TDCs) play a crucial role in ADPLL performance. Yao et al. [19] demonstrated a 0.14 psrms fractional-N digital PLL with an ADC-assisted TDC, significantly improving time resolution. Straayer and Perrott [20] introduced a multi-path gated ring oscillator TDC with first-order noise shaping, offering insights into noise reduction strategies in TDC designs.

Energy efficiency remains a paramount concern for IoT devices. Yang et al. [21] achieved remarkable power efficiency with their 0.2 V energy-harvesting BLE transmitter, demonstrating the potential for near-zero-power wireless communication. Tamura et al. [22] presented a 0.5 V BLE transceiver with excellent power efficiency and interference tolerance, highlighting the importance of low-voltage design techniques. Maintaining spectral efficiency and modulation index stability at high data rates poses significant challenges. Sun et al. [23] demonstrated a compact BLE transmitter with improved system efficiency using duty-cycled edge-timing calibration, addressing some of the challenges in maintaining modulation accuracy at high speeds. Integration and area efficiency continue to be key goals for IoT devices. Wu et al. [24] implemented a wireless connectivity combo IC in 28 nm CMOS, showcasing the potential for integrating multiple wireless standards in a single chip, highlighting the challenges and opportunities in designing multi-standard wireless transceivers for IoT applications.

While significant progress has been made in ADPLL-based GFSK modulators for BLE applications, several areas warrant further investigation. These include achieving small FSK error levels while maintaining high data rates and low power consumption, implementing robust calibration techniques that can adapt to varying environmental conditions in IoT deployments, developing TDC architectures that can provide sub-picosecond resolution without excessive power consumption, designing modulators that can fully comply with the SMI requirements of BLE 5.0 and beyond, and exploring novel architectures that can further reduce power consumption while maintaining or improving performance metrics.

## 3. ADPLL-Based GFSK Modulator Architecture

**Figure 2:**
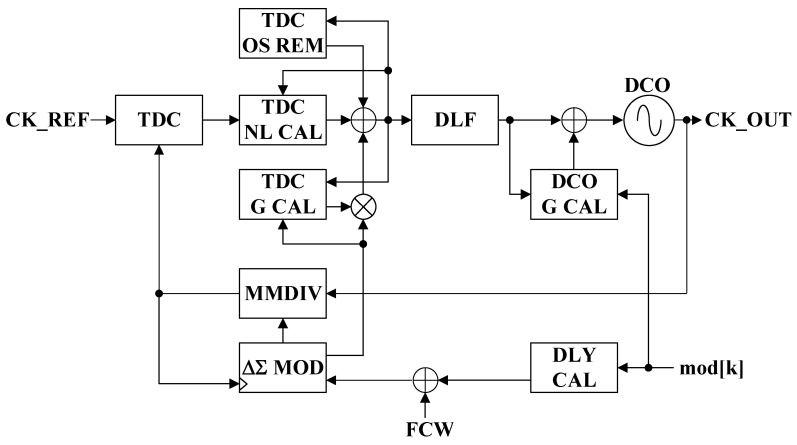
The proposed all-digital phase-locked loop architecture.

The proposed ADPLL architecture is shown in Figure 2. A MASH111 ΔΣ modulator is employed to reduce fractional spurs due to the nonlinearity of a multi-modulus divider (MMDIV) and a TDC. The delay from the DCO clock edge to the feedback clock edge is dependent on the division ratio. The dynamic changes in the MMDIV ratio for the fractional-N ADPLLs result in the increased in-band phase noise floor due to noise folding. This issue is mitigated by using two-stage retiming flip-flops to resynchronize the feedback clock with the DCO clock. The expected phase error is generated from the ΔΣ modulator quantization error. After scaling by the estimated TDC gain calibration (TDC G CAL), the resulting value is subtracted from the TDC output before entering the digital loop filter (DLF). 

The TDC exhibits nonlinearity and small-magnitude phase errors. To improve TDC linearity, a calibration is performed at chip power-up. During this calibration, the ADPLL is set to a selected frequency multiplication ratio to avoid integer boundary values. In addition, the fractional part must have a sufficient Hamming distance to ensure that the phase error sequence is appropriately random [19]. During normal operation, TDC nonlinear calibration (TDC NL CAL) is turned off and the TDC is compensated based on the nonlinearity estimated during calibration. Additionally, the flip-flop that detects phase errors may experience offset and metastability issues. To address these problems when the ADPLL is locked, a TDC offset can be introduced [19]. By adjusting the MMDIV division sequence, phase errors can be shifted away from small magnitude values when the PLL is locked. The digital TDC offset-removal loop (TDC OS REM) monitors the TDC offset magnitude and subtracts the expected offset from the TDC encoder output. This expected offset is generated by applying the correct polarity to the estimated TDC offset magnitude, which can be determined from the anticipated phase error. A low dropout regulator (LDO) is used to provide stable operation of the TDC with respect to process, voltage, and temperature (PVT) variation since the TDC resolution affects ADPLL bandwidth variation.

The phase error output of the TDC is transformed into a frequency tuning word (FTW) in the secondary DLF, and a digitally controlled oscillator (DCO) produces a frequency output signal proportional to the FTW. The proposed ADPLL embeds DCO linearity calibration, delay calibration (DLY_CAL), and DCO gain calibration (DCO_G_CAL) since the two-point modulation is highly sensitive to the DCO nonlinearity, delay mismatch, and gain mismatch for the high data rate modulation.

## 4. ADPLL-Based GFSK Modulator Circuitry

### 4.1. PFD-Based GRO TDC

**Figure 3:**
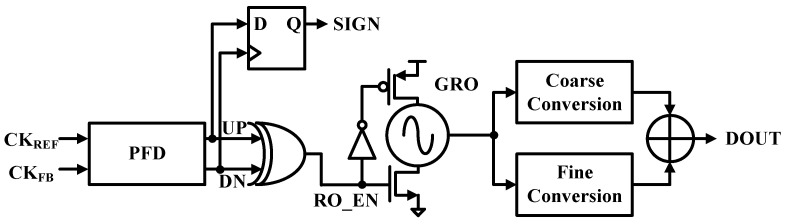
PFD-based GRO TDC.

While conventional gated ring oscillator (GRO) TDCs can cover a wide input range, achieving high resolution is difficult and can be highly dependent on PVT variations. Figure 3 shows a phase frequency detector (PFD)-based gated ring oscillator (GRO) TDC. Up and down signals of the PFD, as described in Section 4.1.1., are connected to an XOR gate and a flip-flop to capture positive and negative phase errors, which have not been captured with conventional TDCs [20]. As described in Section 4.1.2 and Section 4.1.3, the proposed GRO TDC uses a PVT adaptive LDO to make it insensitive to PVT variations. Coarse and fine transforms are used to improve the TDC resolution, as described in Section 4.1.4.

#### 4.1.1. Phase Frequency Detector (PFD)

**Figure 4:**
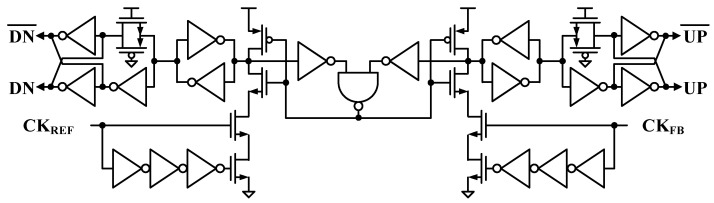
Latch-based PFD with single-to-differential converters.

The PFD employs the three-state machine architecture with the pulsed latches [25], as shown in Figure 4. The PFD reduces the cycle slip which can increase the PLL acquisition time considerably since it does not lose the input clock edge that arrives during the reset period and does not output the wrong direction with a short duration of the reset path. The single-ended-to-differential converter (S2D) is added at the output of the PFD to make differential UP/DN signals for the following CP. The S2D uses a cross-coupled latch that maximizes the output zero-crossing symmetry and is self-compensating over PVT variations, which can reduce the reference spurs of the PLL outputs.

#### 4.1.2. Multipath Gated Ring Oscillator (GRO)

**Figure 5:**
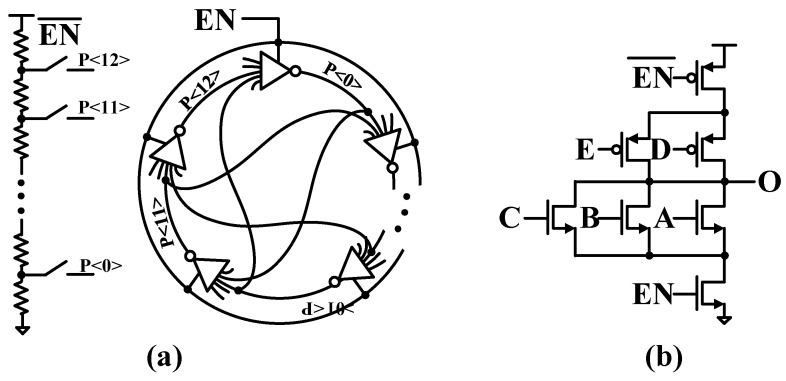
(**a**) 13-stage multi-path gated ring oscillator and (**b**) an interpolated delay cell.

Figure 5a shows a 13-stage multipath gated ring oscillator (GRO) with five inputs using an interpolation delay cell to improve the resolution of one inverter delay cell, as shown in Figure 5b. The interpolation delay cell helps to increase the ring oscillator frequency and prolong the rising and falling transition times of the oscillation waveform. 

In a conventional GRO TDC [20], the GRO holds the final values of each node voltage after oscillation, and the final node voltages will be the start value of the next oscillation cycle. Then, the periodic GRO output can convert any circuit nonidealities into spurs by processing the same input and producing a periodic output during each clock cycle. In the proposed 13-stage interpolated ring oscillator, node voltages are reset to a predefined value derived from a resistor ladder before the TDC starts measuring the phase error, which can eliminate the periodic TDC output signal issue. The GRO also leaks charge while in hold mode, resulting in TDC measurement errors. For fractional-N synthesis applications, the hold time of the node voltages varies with each cycle, and the amount of leakage charge depends from cycle to cycle. The phase initialization makes the GRO start in a coherent state, avoiding the memory of previous conversions.

#### 4.1.3. PVT-Adaptive LDO with Fast Transient Response

The free-running period of the GRO is sensitive to process, voltage, and temperature variations (PVT), which degrades TDC linearity performance. A PVT-adaptive LDO is proposed to provide a near-constant duration of the GRO. The LDO can provide stable supply voltage so that the GRO is insensitive to supply variation. In addition to the conventional LDO, it is required to support process and temperature adaptiveness for highly linear TDC performance. Moreover, the LDO should provide a fast transient response since the GRO runs only during EN assertion time, which is becoming much shorter than the reference clock cycle of the ADPLL when the ADPLL approaches lock status. Moreover, FSK error is increased when the ADPLL is locked and has a short EN pulse assertion. An NMOS LDO can provide a fast settling time due to the small output resistance of NMOS compared to a PMOS LDO [26], but the NMOS gate voltage needs to be higher than the supply voltage because of the NMOS threshold voltage drop, which requires another supply voltage and thick oxide transistors.

**Figure 6:**
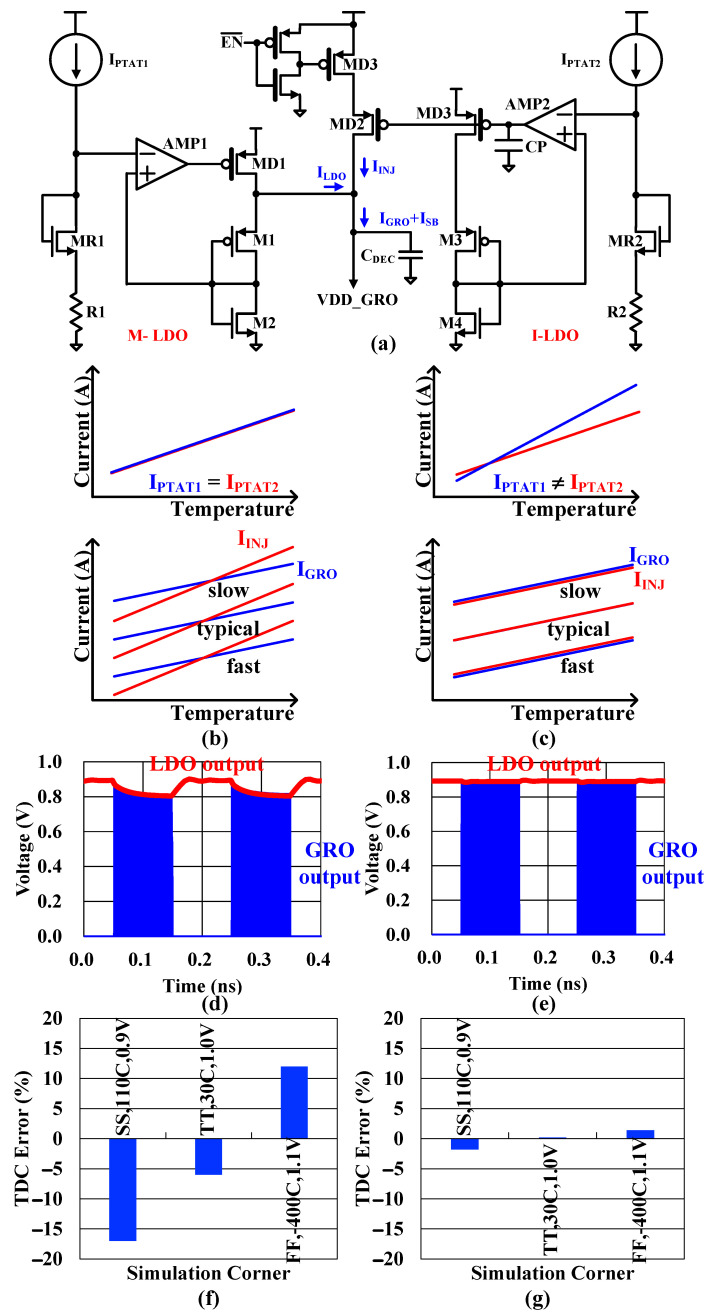
(**a**) PVT-adaptive LDO and PTAT current sources with (**b**) I_PTAT1_ = I_PTAT2_ and (**c**) I_PTAT1_ ≠ I_PTAT2_, simulation results (**d**) without current injection LDO and (**e**) with current injection LDO, and TDC errors due to PVT variations (**f**) without current injection LDO and (**g**) with current injection LDO.

Figure 6a shows the proposed PVT-adaptive PMOS LDO that provides a fast transient response. It consists of a main PMOS LDO (M-DLO) to define the output voltage with serial shunt feedback and a current injection LDO (I-LDO) with a replica bias to compensate for the slow transient response of the M-LDO.

The negative feedback property of the LDOs makes them insensitive to low-frequency supply noise, and the decoupling capacitor (C_DEC_) filters high-frequency noise. The reference circuits of the LDO keep an almost constant free-running frequency of the GRO concerning temperature and process variations.

The reference voltages of both LDOs (M-LDO and I-LDO) are generated by current sources proportional to the absolute temperature (PTAT), I_PTAT_, that require a large saturation voltage to achieve high resistance, which reduces the voltage headroom of the reference circuit. In the proposed LDO, instead of the inverter replica bias circuit consisting of NMOS and PMOS with diodes connected in series, only a diode-connected NMOS bias circuit is used for low-voltage operation, which can compensate for the NMOS process variation of the GRO. The compensation for PMOS process variation is conducted at the feedback path where there is no voltage headroom issue. Even if the proposed LDO uses PTAT current sources, it is difficult to maintain a constant supply voltage of the GRO due to the difference between the current change in the proposed LDO and the current used for the GRO from temperature variation. Figure 6b shows the relationship between the injected current, I_INJ_, the current used by the GRO, I_GRO_, and the output voltage as a function of temperature in the non-optimized case with I_PTAT1_ = I_PTAT2_. At low temperatures, the I_INJ_ is less than the current used by the GRO, I_GRO_, and at high temperatures, the injected current is more than the current used by the GRO. Therefore, a temperature-dependent mismatch occurs, resulting in the RO supply voltage changing during operation, which causes the TDC linearity to deteriorate. On the other hand, if optimized with different slopes of IPTAT_AUX and IPTAT_MAIN, as shown in Figure 6e, the current is tracked against temperature variation to keep the RO supply voltage constant during operation and prevent TDC linearity deterioration. Figure 6b shows the relationship between the injected current, I_INJ_, the current used by the GRO, I_GRO_, and the output voltage as a function of temperature in the non-optimized case with I_PTAT1_ = I_PTAT2_. At low temperatures, the I_INJ_ is less than the I_GRO_, and at high temperatures, the I_INJ_ is more than the I_GRO_. Therefore, a temperature-dependent mismatch occurs, resulting in the GRO supply voltage changing during operation, which causes the TDC linearity to deteriorate. To overcome this, resistors R1 and R2 are connected in series with two reference circuits and use different resistor values to optimize the slope of I-LDO’s change with the temperature change to be smaller than M-LDO so that the power supply voltage of the GRO can be kept constant during operation and to prevent TDC linearity deterioration through the LDO adapting to temperature variation. Figure 6c shows the relationship between the I_INJ_, the I_GRO_, and the output voltage as a function of temperature in the optimized case with I_PTAT1_ ≠ I_PTAT2_, which enables the I_INJ_ to track against temperature variation to keep the GRO supply voltage constant during operation and prevent TDC linearity deterioration.

It also provides a fast transient response with $\bar{EN}$ equal to the GRO EN signal assertion time, and the injection current compensates for the sudden load current changes caused by GRO oscillations. Figure 6d shows the output waveforms of the LDO and GRO with only the M-LDO. The M-LDO starts to drop the output voltage during the GRO oscillation because the M-LDO bandwidth is not enough to recover the output level during the short duration of the EN signal. Figure 6e shows that there is little voltage fluctuation thanks to the I-LDO injection current, even when the GRO starts to oscillate abruptly, which enhances the TDC linearity performance and decreases the FSK error. Figure 6f,g show the TDC errors due to PVT variations with and without the current injection LDO, which verifies that the proposed LDO with the injection current LDO have 1.4% TDC error compared to 18% error without the injection current LDO.

#### 4.1.4. Coarse and Fine Conversions

**Figure 7:**
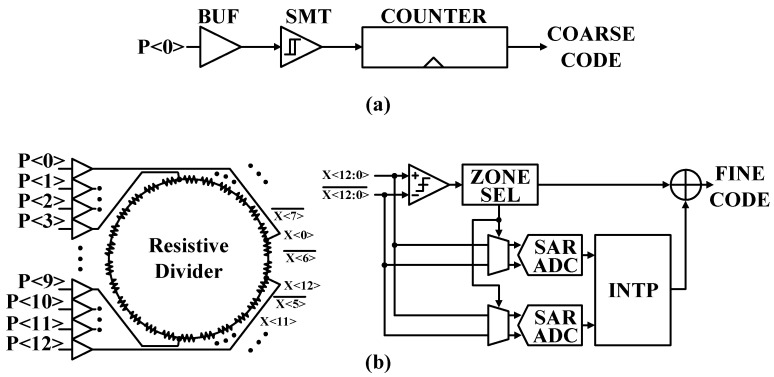
(**a**) Coarse conversion and (**b**) fine conversion TDCs.

The GRO starts to oscillate and accumulate phase error when the input signal EN is asserted, as discussed in Section 4.1.1. The GRO output goes through a Schmitt trigger to obtain a square wave that drives a counter for a coarse conversion result, *T_coars_* as shown in Figure 7a.

The fine converter shown in Figuiire 7b uses an analog-to-digital converter (ADC)-assisted TDC to improve TDC resolution by converting a pair of residue voltages from the coarse stage with a pair of 7-b successive approximation (SAR) ADCs [19]. The 26 resistors connected in a ring type are tethered to the GRO output node of each node to create a differential signal to the GRO output through voltage division, as X<12:0> and $\bar{X<12:0>}$. The 13-zone selector (ZONE SEL) defines the phase of the GRO to within 1/13 of the GRO period. Then, a pair of nodes near the low-to-high zero-crossing point, *V_L_* and *V_H_*, are selected and digitized with the SAR ADCs. The digital interpolator (INTP) estimates an almost zero-crossing point, and the fine conversion result, *T_fine_*, can be expressed as Equation (2).

(2) $$T_{fine}=\left(\frac{V_{L}}{V_{H}-V_{L}}\right)T_{coarse}\;$$

The effective TDC resolution, *T_res_*, can be estimated as Equation (3).

(3) $$T_{res}=\frac{T_{GRO}}{N}\left(\frac{1}{V_{H}-V_{L}}\right)\;$$

where TGRO is the GRO free-running period, *N* is the number of stages in the GRO, and *V_H_* − *V_L_* is the average digital code difference between the two SAR ADCs in the fine conversion, which makes the TDC resolution equal to sub-picosecond resolution.

### 4.2. LC DCO with ΔΣ Modulator

**Figure 8:**
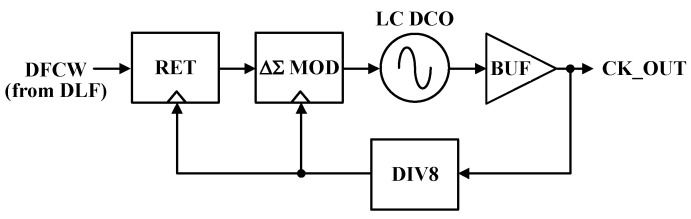
CMOS LC DCO with ΔΣ modulator and retiming.

The LC DCO is preferred for high-frequency WSNs since it provides low phase noise and high frequency stability despite the large size and high cost compared to the ring DCO. Figure 8 shows the LC DCO with the ΔΣ modulator (ΔΣ MOD) and retiming. Frequency dithering is applied to reduce DCO quantization noise with a divide-by-8 DCO (DIV8) clock. The DLF’s input digital frequency control word (DFCW) is synchronized to the ΔΣ MOD using the DIV8 clock in the re-timer (RET). The second-order ΔΣ MOD shapes the DCO quantization noise to a high offset frequency to meet LO phase noise requirements for the WSN applications. Moreover, the dithering technique enhances the frequency resolution of the DCO. Furthermore, 8-bit fine-tuning cells are arranged in a 16 × 16 array, and one varactor fine-tuning cell is variable with 4-bit DAC control.

#### 4.2.1. LC DCO with Coarse and Fine Capacitive Banks

**Figure 9:**
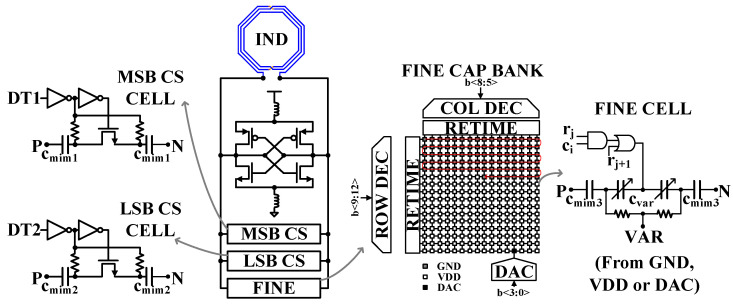
LC DCO with coarse and fine capacitive banks.

In LC-based oscillator design, a CMOS complementary architecture, as shown in Figure 9, is typically used for low power consumption. This architecture features doubled transconductance (*g_m_*) due to the cross-coupled NMOS and PMOS pairs. It is crucial to maximize the inductance (*L*) and quality factor (Q-factor, *Q_L_*) of the DCO, as the minimum oscillation condition of transconductance is defined by Equation (4).

(4) $$L>\frac{1}{2\pi f_{c}Q_{L}g_{m}}\;\;$$

where *f_c_* is oscillation frequency.

The LC DCO consists of three capacitor banks, i.e., the most significant bit coarse (MSB CS), the least significant bit coarse (LSB CS), and fine banks. All capacitor banks were designed with thermometer-weighted instead of binary-weighted structures to ensure linearity in the frequency step. The proposed DCO employs 24 capacitors, which is not a binary number in the MSB bank. Furthermore, 31 LSB capacitors are adopted in the LSB bank for 5-bit control. The unit switch capacitor cell of the coarse capacitive banks consists of two inverters, two resistors, and an NMOS switch, as shown in Figure 9. In the ON mode, the gate voltage of the switch is VDD, and the source voltage is zero. Otherwise, in the OFF mode, the gate voltage is zero, and the source voltage is VDD. Therefore, the switch is in linear region operation in the ON mode and maintains off-state in the OFF mode even with a DCO large swing. The fine-tuning bank is implemented using 12-bit control bits, as illustrated in Figure 9. The eight MSBs are organized into a 16 × 16 array of 1 MSB fine-tuning cells. In this array, all elements except one are connected either to VDD (gray cells) or to ground (white cells), creating a thermometer coding pattern in the matrix. The remaining varactor (black cell) is connected to the output of a 4-bit digital-to-analog converter (DAC), providing 16 additional voltage levels between VDD and ground. Because only one varactor is biased at the point of its characteristic with high voltage-to-frequency gain, the oscillator’s sensitivity to noise and spurious signals coupled at the DAC output is minimized. While quantizing the varactor characteristic is not strictly necessary to achieve the target fine frequency resolution, this approach simplifies the matrix routing.

#### 4.2.2. GFSK Frequency Control Word (FCW) Range of DCO

**Figure 10:**
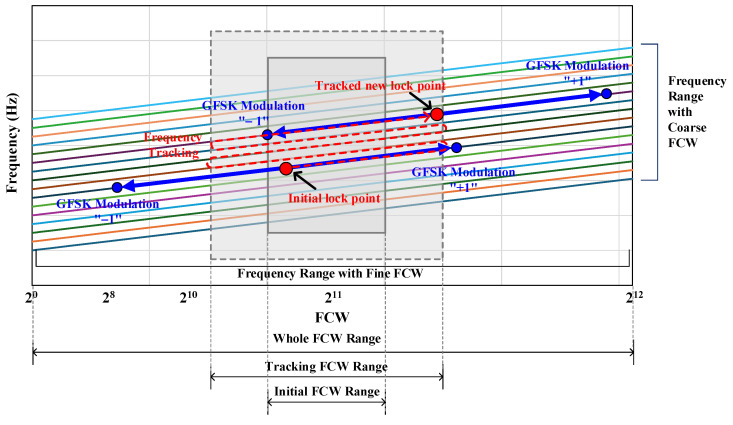
Frequency range of the DCO with coarse- and fine-tuning frequency control word.

The ADPLL uses a negative feedback mechanism to find the locked state. Figure 10 shows the behavior of GFSK modulation with frequency control words (FCWs) over the coarse- and fine-tuning capacitor bank frequency range. The fine-tuning capacitor bank can cover more than 2 MHz. For the initial lock operation, the frequency control words (FCWs) of the fine-tuning capacitor bank are limited to within ±250 kHz centered at 2^11^ FCWs. After the initial lock, the GFSK modulation of ±500 kHz centered on the initial lock point is performed for a 2 Mb/s data rate. For BLE operation, the ADPLL continuously tracks new lock points to adapt to environmental changes. For the tracked new lock point, the FCW of the fine-tuned capacitor bank is limited to within ±500 kHz centered on 2^11^ FCWs, and the GFSK modulation of ±500 kHz centered on the tracked new lock point is performed.

### 4.3. LMS-Based DCO Gain Calibration

Two-point direct frequency modulation (FM) is applied to the DCO frequency control and the MMDIV ratio control when the ADPLL is locked to the desired channel frequency, as shown in Figure 2. The phase mismatch is calibrated by adjusting the modulation data delay at the MMDIV (DLY CAL). The DCO gain calibration requires an accurate estimation of the DCO gain, K_DCO_, which is nonlinear and highly sensitive to PVT variations. A least mean square (LMS)-based gain calibration for the DCO accurately tunes the gain of the oscillator to ensure its output frequency closely matches the desired frequency. The LMS algorithm is an adaptive method that iteratively adjusts the control parameters to minimize the error between the actual and target frequencies. Previous work [18] updates the correction every time the FM data crosses zero; therefore, if the number of FM data zero-crossing events is small or the data rate is low, the correction will take a long time or the FSK error will not converge within a certain range. Also, in previous work, the amount of correction at every event is proportional to the amount of filtered phase error (ϕ_E_), so, if the frequency error is large, the correction process is fast, but if the ϕ_E_ starts to converge to zero, it takes a long time to minimize the FSK error.

**Figure 11:**
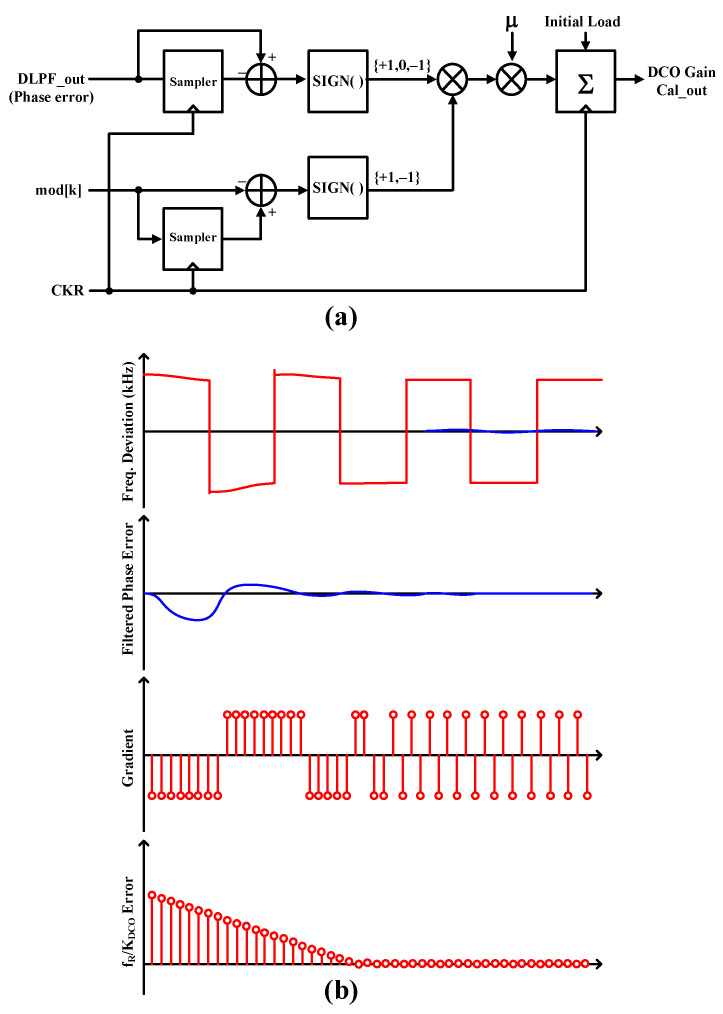
(**a**) LMS adaptation algorithm for calibrating the DCO gain and (**b**) DCO gain adaptation process example.

Figure 11a shows the proposed LMS-based DCO gain calibration. It provides a fast calibration approach compared to previous work that updates for zero-crossing events in the FM data since the calibration occurs at every reference clock cycle (*f_R_*). Furthermore, since the proposed LMS utilizes only the sign information of the phase error and the sign information of the modulation data fluctuations, the gain error converges linearly with the predefined adaptation step size (*μ*), decreasing by a constant correction amount in each cycle, as shown in Figure 11b. Therefore, the gain error with the proposed LMS calibration can approach almost zero error faster than the previous work that utilizes the proportionality of the filtered phase error. The updated value of the DCO gain calibration output, *f_R_*/*K_DCO_*, is given by Equation (5).

(5) $$\frac{f_{R}}{K_{DCO}}\left[n\right]=\frac{f_{R}}{K_{DCO}}\left[n-1\right]+\mu \cdot \mathrm{sign}\left(\phi_{E}\right)\cdot \mathrm{sign}\left(mod\left[n\right]-mod\left[n-1\right]\right)$$

The calibration process operates continuously in the background. For each transmitted data packet, the system updates the initial value using the most recent gain value determined by the LMS calibration algorithm.

## 5. Experimental Results

**Figure 12:**
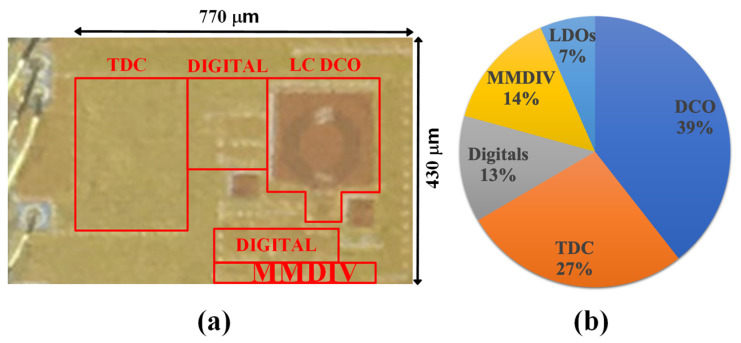
(**a**) Microphotograph and (**b**) power breakdown of the proposed GFSK modulator based on ADPLL.

Figure 12a shows a micrograph of the proposed GFSK modulator implemented in a 1P8M 28 nm CMOS process. It includes the ADPLL with a TDC, an LC DCO, an MMDIV, digital blocks for a DLF, and digital controllers. The TPM is applied to the ADPLL to achieve 2 Mb/s GFSK with 32 MHz CK_REF. The core area is 0.331 mm2. A 1.0 V power supply is used, and the total power consumption is 1.6 mW. Figure 12b shows power breakdown. The DCO, TDC, MMDIV, digitals, and LDOs consume 633 μW, 436 μW, 227 μW, 204 μW, and 106 μW, respectively. The GFSK modulator design demonstrates a careful balance between power consumption, linearity, and frequency range for BLE applications. The modulator achieves an excellent linearity of 1.42% FSK error while consuming only 1.6 mW, covering a wide frequency range of 2.21–2.58 GHz. There are trade-offs to consider. Improving linearity further would likely increase power consumption. Reducing the frequency range could potentially lower power consumption but might compromise robustness to PVT variations. The current design appears well optimized for BLE, but further refinements could be made depending on specific application priorities.

**Figure 13:**
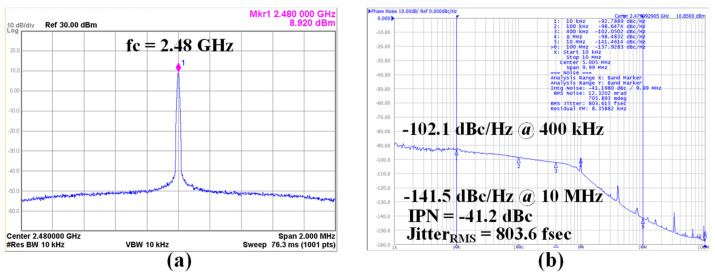
(**a**) ADPLL spurious performance and (**b**) phase noise at 2.48 GHz.

The ADPLL spectrum output at 2.48 GHz is shown in Figure 13a, and there is no noticeable spurious tone in the 2 MHz bandwidth. The phase noise of the ADPLL was measured at 2.48 GHz with a frequency multiplication ratio of 77.5, as shown in Figure 13b. This is the high-end center frequency of the BLE bands. The digital loop filter bandwidth is 750 kHz. The in-band phase noise measured at a 400 kHz offset is −102.1 dBc/Hz. The out-of-band phase noise at a 10 MHz offset is −141.5 dBc/Hz. The integrated phase noise from 100 k to 10 M is −41.2 dBc, and the root mean square (RMS) jitter is 803.6 fsec. The ADPLL can support from 2.21 to 2.58 GHz to cover whole BLE bands with margin.

**Figure 14:**
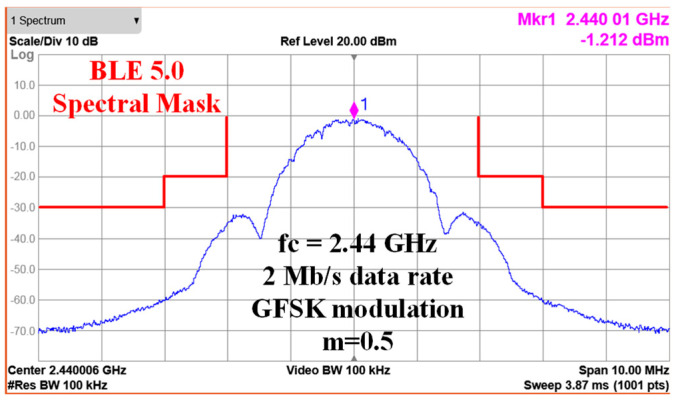
GFSK modulation spectrum at 2.44 GHz.

**Figure 15:**
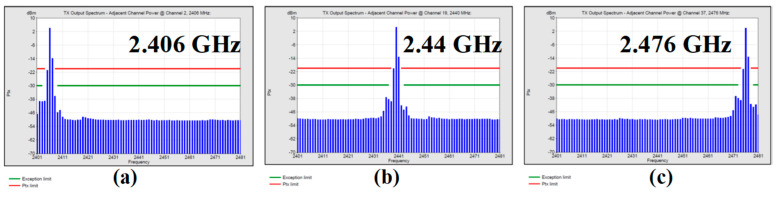
2 Mb/s GFSK spectrums at (**a**) 2.406 GHz, (**b**) 2.44 GHz, and (**c**) 2.476 GHz.

Figure 14 shows the GFSK modulation spectrum of the BLE mode at 2.44 GHz. The modulated output spectrums are measured with the maximum Pout setting and a 2^31^ − 1 pseudorandom binary sequence (PRBS). The data rate of the BLE mode is 2 Mb/s with a modulation index of 0.5. The side lobe is lower than the main lobe by about 30 dB. Figure 15 shows the GFSK spectrums at 2.406, 2.44, and 2.476 GHz, respectively. All spectrums meet the BLE 5.0 spectral mask requirement fully.

**Figure 16:**
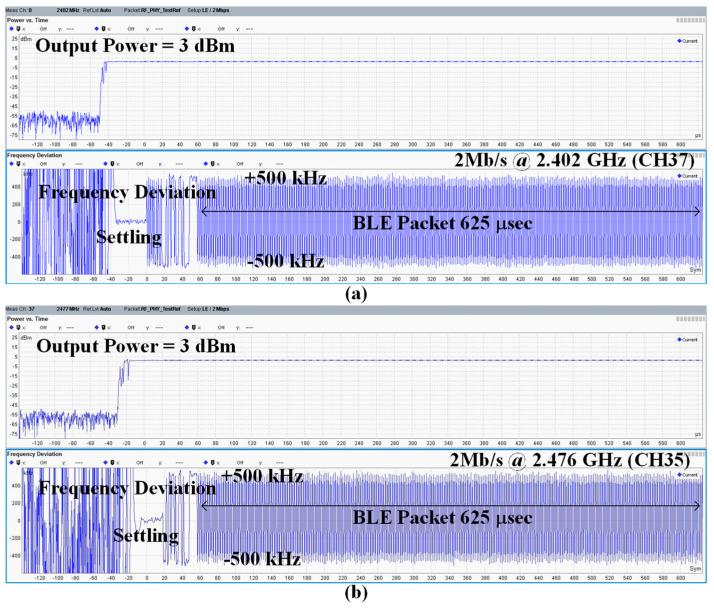
BLE output power and demodulated frequency for the BLE packet at (**a**) 2.402 GHz and (**b**) 2.477 GHz.

Figure 16 shows the performance of a 2 Mb/s BLE packet transmission on advertising channel 37 (f_CH_ = 2.402 GHz) and data channel 35 (f_CH_ = 2.476 GHz). The maximum difference between the 0/1 symbol frequency at the start of a BLE packet and the 0/1 frequency within the packet payload 625 μs is less than ±50 kHz, which is the BLE specification. This specification is adequately met with a margin of more than an order of magnitude even during open-loop operation. In addition, the ADPLL settling time is about 10 μs.

**Figure 17:**
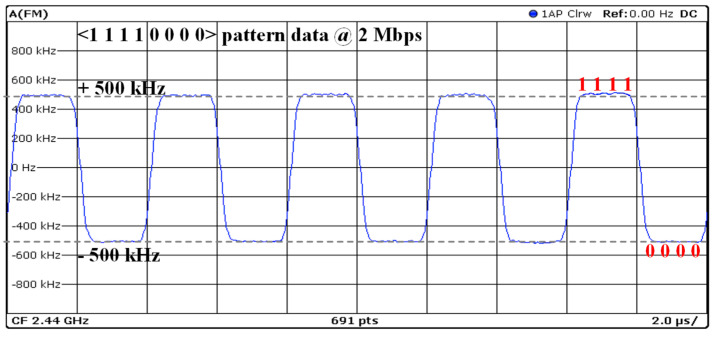
Frequency deviation for stable modulation index (SMI) with 1111000 data.

The BLE 5.0 specification defines a stable modulation index (SMI) between 0.495 and 0.505 in the <00001111> continuous test pattern at a 2 Mb/s data rate. Figure 17 shows the frequency deviation to check the SMI at the center frequency of 2.44 GHz. The average deviation is 0.503, which fully meets the SMI specification.

**Figure 18:**
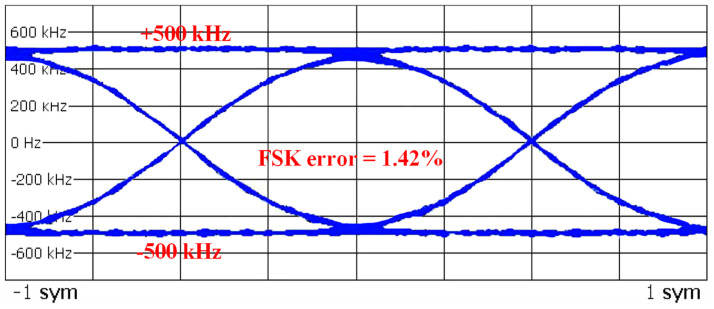
Eye diagram of 2 Mb/s GFSK data.

Figure 18 plots the eye diagram normalized at 500 kHz for 2 Mb/s GFSK modulation. The 1.42% FSK error was achieved due to the very high linearity of the TDC and DCO and the LMS-based DCO gain compensation for the TPM. Table 1 summarizes the performance of the proposed work and its comparison with the state-of-the-art PLL-based GFSK modulator [14,15,16,17,21,22,23,24]. The proposed GFSK modulator supports three advertising channels and 37 data channels from 2.4 to 2.48 GHz. Compared to the state of the art, the proposed modulator provides the maximum data rate of 2 Mb/s with TPM and the best in-band phase noise performance of −102.1 dBc/Hz. Moreover, the proposed modulator supports the best FSK error of 1.42% and full SMI specifications of BLE 5.0 standards by using PVT adaptiveness and linearity background calibrations for a TDC and a DCO.

## 6. Conclusions

The ADPLL-based BLE 5.0 2 Mb/s data rate GFSK modulator is demonstrated in 28 nm CMOS technology with an area of 0.33 mm^2^. A TPM is applied to overcome the bandwidth limitation of the ADPLL. The proposed ADPLL utilizes a PVT-adaptive LDO and a hierarchical conversion with coarse and fine time resolution to achieve high time resolution and highly linear TDC performance. The fine resolution is supported by a 7-bit SAR ADC to achieve sub-picosecond resolution. DCO linearity is improved by using dithering with a ΔΣ modulator. DCO linearity is also improved by a 12-bit fine-tuned capacitor bank consisting of a 16 × 16 array of the same unit cell size for the MSB and a 4-bit digital varactor for the LSB. A hierarchical coarse-tuning capacitor bank of 24 coarse-tuning capacitors for MSB and 31 coarse-tuning capacitors for LSB, each with a thermometer-weighted cell, is added to achieve a wide tuning range that covers 40 BLE bands from 2.4 to 2.48 GHz with sufficient margin. The proposed LMS-based DCO gain compensation helps to meet the stable modulation index (SMI) specification. The proposed modulator demonstrates 2 Mb/s data rate GFSK modulation with TPM over a wide frequency tuning range from 2.21 GHz to 2.58 GHz. The highly linear ADPLL provides 1.42% FSK error while consuming 1.6 mW. With full on-chip integration and high efficiency, the proposed GFSK modulator complies with BLE 5.0 requirements, enabling long-life IoT sensor nodes that ubiquitously support wireless personal area networks (WPANs) and wireless body area networks (WBANs). Compared to technologies like ZigBee or earlier BLE versions, it provides better spectral efficiency and higher data rates while maintaining competitive power consumption. These features make it particularly suitable for applications requiring higher throughput or improved energy efficiency. However, real-world performance evaluation is crucial, considering factors such as range, interference resilience, and integration challenges. While the modulator shows potential for enhancing WSN performance, comprehensive field testing is necessary to fully validate its advantages in actual deployments. Future work could improve the modulator to support other wireless sensor network standards without major modifications, as the computational optimization of the modulator proposed for BLE 5.0 may limit its flexibility.

## Figures and Tables

**Table 1:** Performance summary of the proposed GFSK modulator with PLLs and comparisons to the state of the art.

	This Work	[16] TCAS-I 22	[14] JSSC 21	[23] ESSCIRC 21	[17] RFIC 20	[22] JSSC 20	[15] JSSC 19	[21] JSSC 19	[24] RFIC 18
Technology (nm)	28	40	40	65	65	65	40	22	28
PLL Architecture	ADPLL	ADPLL	ADPLL	ADPLL	BB-DPLL	ADPLL	DPLL	Analog PLL	ADPLL
Oscillator Type	LC DCO	LC VCO	LC DCO	Ring DCO	LC DCO	LC DCO	Ring VCO	LC VCO	LC DCO
Frequency Range	2.21~2.58	2.36~2.40	2.1~2.7	2~2.8	1.6~1.94	2.4~2.48	2.4~2.48	2.4~2.48	2.4~2.48
GFSK Modulation	Two-Point	Two-Point	Two-Point	Single Point	Two-Point	Two-Point	Single Point	Single Point	Two-Point
Data Rate (Mb/s)	2	0.971	2	2	1	2	1	1	1
Background Calibration	Yes	Yes	Yes	Yes	No	No	Yes	Yes	Yes
In-Band Noise (dBc/Hz)	−102.1 (@2.48 GHz)	−94 (@2.4 GHz)	N/A	−85.7 (@2.4 GHz)	−98 (@1.2 GHz)	N/A	−85 (@2.402 GHz)	−83 (@2.428 GHz)	N/A
Power (mW)	1.6	4.85	1.96	1.2	5.3	7.8	1.55	3.97	N/A
FSK Error (%)	1.42	2.3	1.86	5.9	2.86	1.6	9.1	2.84	N/A
SMI	Yes	No	No	No	No	No	No	No	Yes
Core Area (mm^2^)	0.331	0.48	0.38 *	0.25	0.383	0.63	0.017	0.53	1.47 *

* Estimated area from chip microphotograph.

## Data Availability

Data are contained within the article.
